# Exposure to the UV Filter Octyl Methoxy Cinnamate in the Postnatal Period Induces Thyroid Dysregulation and Perturbs the Immune System of Mice

**DOI:** 10.3389/fendo.2019.00943

**Published:** 2020-01-31

**Authors:** Fausto Klabund Ferraris, Esdras Barbosa Garcia, Amanda da Silva Chaves, Thais Morais de Brito, Laís Higino Doro, Naína Monsores Félix da Silva, Amanda Soares Alves, Tatiana Almeida Pádua, Maria das Graças M. O. Henriques, Tiago Savignon Cardoso Machado, Fabio Coelho Amendoeira

**Affiliations:** ^1^Laboratory of Pharmacology, Department of Pharmacology and Toxicology, National Institute of Health Quality Control (INCQS)—Oswaldo Cruz Foundation (FIOCRUZ), Rio de Janeiro, Brazil; ^2^Laboratory of Applied Pharmacology, Institute of Drug Technology (Far-Manguinhos)—Oswaldo Cruz Foundation (FIOCRUZ), Rio de Janeiro, Brazil; ^3^Laboratory of Professional Education in Laboratory Techniques in Health, Polytechnic School of Health Joaquim Venâncio—Oswaldo Cruz Foundation (FIOCRUZ), Rio de Janeiro, Brazil

**Keywords:** sunscreen, UV filter, thyroid disruptor, hypothyroidism, octyl methoxycinnamate, OMC, immune system

## Abstract

Evidence demonstrates the bidirectional communication and regulation between the neuroendocrine and immune systems. Thyroid hormones play key roles in nervous system development and can exert influence on various immune cells contributing to pathophysiological conditions. Octyl methoxycinnamate (OMC) is one of the most commonly used UV filters, and *in vitro* and *in vivo* studies have found thyroid disrupting effects. The present study assessed whether OMC administration in mice dams during the lactational period can cause thyroid disruption and generate immunologic alterations in the offspring. Indirect exposure to the OMC (1,000 mg/kg) in the lactational period affected neurodevelopment parameters, such as delayed eye-opening and weight gain in mice of both sexes, and these alterations are corroborated by the decrease in the T4 levels present in the pups' blood. No significant changes were observed in the thymus of these pups, but the number of lymphocytes increased in the spleen of the animals exposed to OMC, similar to the animals treated with propyl-thiouracil (PTU), a well-known thyroid disruptor. OMC modulated the percentage of leukocyte populations in peripheral blood, and the number of circulating polymorphonuclear cells increased two-fold. *In vitro*, OMC exhibited an inhibitory effect on splenocyte proliferation and IL-2 production induced by anti-CD3 antibody; however, this effect was reversed with the addition of T4 in the cell culture. In summary, the results of the present study demonstrate the influence of OMC on thyroid dysregulation and its impact on the modulation of the immune system in mice pups.

## Introduction

The octyl methoxycinnamate (OMC), also known as octinoxate, is probably the organic ultraviolet (UV) filter used most by the cosmetic industry. UV filters, such as OMC, can be bioaccumulated in organisms due to their high lipophilicity and poor degradability (1–5); therefore, they have become contaminants of emerging concern (5). Studies have previously reported that OMC can penetrate through the epidermis and the dermis, spread through the systemic circulation and can have a systemic action on the body, due to its relatively low molecular weight, and lipophilic character (6). Consequently, OMC has been detected in human bodily fluids such as urine and blood after topical application (7). OMC was reported to induce acute toxicities, and a large number of studies, both *in vivo* and *in vitro*, found multiple endocrine disrupting effects in the estrogen receptor (ER), androgen receptor (AR), progesterone receptor (PR), and hypothalamus-pituitary-thyroid (HPT) axis (8–10).

Several UV filters have already been cataloged and reported as a HPT function deregulators, especially when exposed to during the early stages of development (11). These actions can directly affect the gland and/or the corresponding regulatory centers, such as the hypothalamus and the pituitary, affecting the levels of thyrotropin releasing hormone (TRH) and/or thyroid-stimulating hormone (TSH), which are directly related to the synthesis of thyroid hormones. Most studies have focused on the estrogenic and anti-androgenic effects of OMC in wild and lab animals (12–14); however only a few studies focused on the influence of OMC upon the HPT function (15–17).

Several studies have demonstrated the importance of thyroid hormones in ontogenesis, acting on embryonic and fetal tissues, via active transport of maternal thyroid hormone across the placenta to ensure normal development, until the fetal thyroid gland reaches maturity (18). Thyroid hormones are an important coordinators of embryonic and early postnatal development, conducing the metabolism, thermogenesis, the stimulation of growth and the development of various tissues; thus, abnormalities of thyroid hormone levels in infancy and childhood may result in dysfunctional effects in adults. Alterations of the thyroid function can also affect the immune system. Most studies assessing the interrelationship between thyroid and immune system are based on pathophysiological models where the immune system is already altered, such as autoimmune thyroid diseases (e.g., hyperthyroidism—Grave's Disease and hypothyroidism—Hashimoto's thyroiditis) (19). Different studies have generally shown that hyperthyroidism increases the immune response, antibody production, cell proliferation and migration, reactive oxygen species production, and downmodulation of proinflammatory markers (20–22). On the other hand, cases of hypothyroidism produce antagonistic effects on parameters of the immune function; such as decreased immune response, lower antibody production, and perturbed migratory and proliferative capacity of immune cells (23, 24).

The sensitivity of immune system to thyroid disruptor compounds is poorly explored in the neonatal period, added to the fact that the effects of chemicals may be different when administered to adults and neonates; therefore, the present study assessed whether OMC administration in mice dams during the lactational period can cause thyroid disruption and generate immunologic alterations in the offspring.

## Materials and Methods

### Materials

Phosphate buffered saline (PBS), ethylenedyaminetetracetic sodium salt (EDTA), HEPES, bovine serum albumin (BSA), RPMI 1640 and Hank's balanced salt solution (HBSS) were purchased from Sigma-Aldrich (St. Louis, MO, USA). The 6-propyl-2-thiouracil (PTU) and 2-ethylhexyl-4-methoxycinnamate (Octyl Methoxycinnamate—OMC) were purchased from Sigma-Aldrich (St. Louis, MO, USA). IgG anti-murine CD3 (clone 145-2C11), APC-conjugated hamster IgG anti-murine CD3, PE-conjugated hamster IgG anti-murine CD8, and FITC-conjugated rat IgG anti-murine CD4 were all obtained from EXBIO Praha (Vestec, Czech Republic). Fetal bovine serum was obtained from Hyclone (Logan, UT, USA). Carboxyfluorescein diacetate-succinimidyl ester (CFSE) was obtained from Invitrogen (Carlsbad, CA, USA). Cell Proliferation Kit I (MTT) was purchased from Sigma-Aldrich (St. Louis, MO, USA). Thyroxine (T4) AccuBind® kit was purchased from Monobind Inc. (Lake Forest, CA, USA).

### Mouse Line and Animal Care

The Swiss Webster mice used in this study were provided by the Oswaldo Cruz Foundation breeding unit (Rio de Janeiro, Brazil). The animals were kept under standard laboratory conditions, with free access to food and fresh water in a room with the temperature ranging from 22 to 24°C and a 12 h light/dark cycle. The animals were housed at the INCQS experimental animal facility unit until use. After a 3-days cohabitation period, females were removed from the male's cage, and housed individually. Starting on gestational day 15, the cages were daily checked for deliveries. On post-natal day (PN) 1, pups were sexed and allocated into experimental groups. Twenty-four hours after birth pups were divided into two groups (i.e., 4 males and 4 females). Pups were kept with theirs respective mothers inside individual standard plastic cages with stainless steel coverlids and pinewood shavings as bedding. All experimental procedures were performed according to The Committee on Ethical Use of Laboratory Animals of the Fundação Oswaldo Cruz (FIOCRUZ, Brazil; license LW-30/14).

### Post-natal Development, Weight Gain, and Weaning

To evaluate the subacute toxicity of the OMC during the lactation period and to define the dose to be used in subsequent trials, lactating female mice were exposed to different doses of OMC (250, 500, or 1,000 mg/Kg/day), PTU—a known thyrotoxic compound (4 mg/Kg/day) or corn oil (vehicle) by gavage for 22 days (9). One day after birth, the offspring was randomized again into three experimental groups designating one couple/group. The lactating female mice received OMC, PTU or corn oil (vehicle) during the lactational period (PN1 to PN22) daily at 10 a.m. by gavage. To investigate the influence of OMC on their development, pups were observed from PN1 to PN16 to evaluate developmental parameters such as the eruption of the incisors, hair growth and opening of eyes (exact day). On PN23 the Swiss Webster pups, as well as lactating female mice, were euthanized and organs such as thymus and spleen were carefully collected, weighed and processed for cell count and flow cytometry analysis. The peripheral blood from these animals was also collected for leukocyte count and hormone dosage.

### Measurement of Total T4 Hormone Serum Levels

Total T4 was determined in mouse serum by enzyme immunosorbent assay (EIA) Thyroxine (T4) AccuBind® kit following the instructions of the manufacturer (Monobind Inc., Lake Forest, CA, USA). Absorbance was read at 450 nm using a Spectramax M5 microplate reader (Molecular Devices, Sunnyvale, CA, USA).

### Cell Counts

Total cell counts from the thymus, spleen and peripheral blood were conducted using a Neubauer chamber, under an optical microscope, after dilution in Turk fluid (2% acetic acid). The thymus and spleen cell counts are reported as the number of cells per gram of tissue. The peripheral blood counts are expressed as cells per milliliter.

### MTT-Based Proliferation Assay

The splenocyte proliferation was measured by the Cell Proliferation Kit I (MTT) of Sigma-Aldrich (St. Louis, MO, USA) according to the manufacturer's protocol. Splenocytes, recovered from 3 week old male Swiss mice, were treated for 1 h with different concentrations of OMC (1–200 μg/mL) or vehicle; and subsequently cultured in the anti-CD3 mAb-coated wells at a concentration of 10^5^ cells/well in RPMI 1640 medium supplemented with 10% FBS (at 5% CO_2_ and 37°C). After 72 h, the cells were incubated with the MTT solution for another 4 h. The water insoluble formazan dye was solubilized before the measurement of absorbance using a Spectramax M5 multiwell spectrophotometer (Molecular Devices, Sunnyvale, CA, USA). The absorbance was read at 550 nm (25).

### CFSE-Based Proliferation Assay

Splenocytes, recovered from 3 week old male Swiss mice, were labeled with the cell proliferation dye carboxyfluorescein diacetate succinimidyl ester (CFSE) kit (Invitrogen, Carlsbad, CA, USA) according to the manufacturer's protocol and stimulated for 72 h with anti-CD3 antibody after treatment for 1 h with OMC, T4 (10^−5^ M), OMC plus T4, or vehicle. Proliferation was assessed by the percentage of CFSE+ high cells (0 h) compared to CFSE+ low cells (72 h) as analyzed by CyFlow Space flow cytometer (Partec GmbH—a Sysmex Company, Münster, Germany) (26).

### Enzyme-Linked Immunosorbent Assay (ELISA)

The concentrations of IL-2 in the supernatants from the proliferation assay were evaluated by sandwich ELISA using matched antibody pairs (Quantikine, R&D Systems, Minneapolis, MN, USA) according to the manufacturer's instructions. The results are expressed as nanograms per milliliter (ng/mL) (26).

### Statistical Analysis

All data distributions were used to check normality by Kolmogorov-Smirnov test. In this case, a value of *p* > 0.1 suggests normal distribution. For the data with normal distribution, mean and standard error of the mean (SEM) were calculated. The treatment groups were compared by Student's *t*-test for independent samples. For the evaluation of weight gain, we used ANOVAr (ANOVA repeated measure), with sex and group like between-subject factors. For data with free distribution, proper statistical analyses were performed for each type of data. The variables: ear detachment, hair growth, eruption of the incisors, and opening of eyes were analyzed by Chi-Square test. For all tests, a value of *p* < 0.05 was considered as statistically significant. The statistical analyses were created using Graph Pad Prism Program 3 version 2.01 and SPSS Program version 15.0 for Windows.

## Results

**Figure 1:**
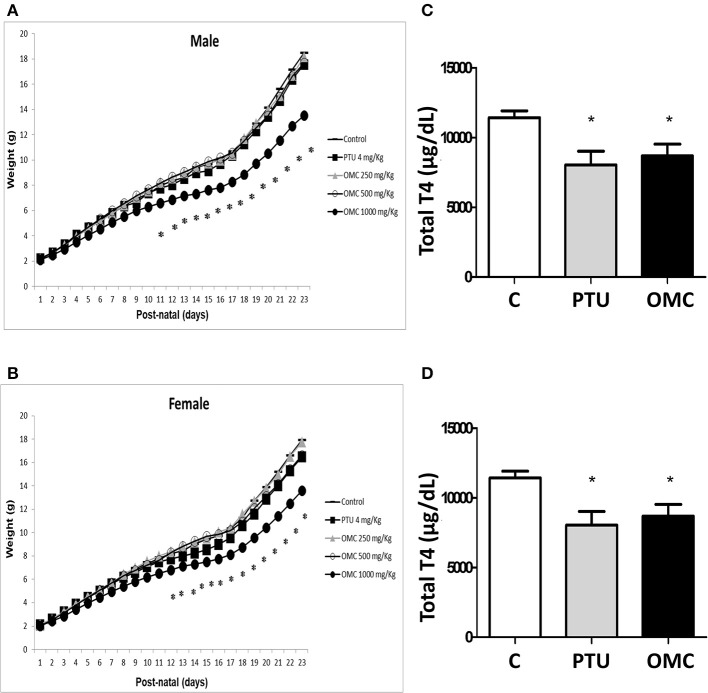
Weight gain and measurement of total T4 hormone in animals exposed to PTU or OMC. Weight gain of **(A)** male and **(B)** female pups following exposure of lactating female mice to different concentrations of OMC. **(C)** Total T4 hormone serum levels in pups and **(D)** lactating female mice on PN23. **(A)** (*N* = 17); **(B)** (*N* = 17); **(C)** (*N* = 13); **(D)** (*N* = 13); *Significant difference between exposed groups and Control (*p* < 0.05). Error bars correspond to ± SEM.

Lactating pups fed by females exposed to 1,000 mg/Kg/day of OMC showed a significant reduction in weight gain and a delay in eye opening compared to the vehicle group (Figures 1A,B and Table 1). These data were in agreement with results obtained by our group in rats (unpublished data), where the direct exposition to OMC interfered in diverse developmental parameters in pups, wich were linked to a decrease of thyroid activity. To test whether the observed alterations in the offspring could have any correlation with thyroid disruption, the plasma T4 levels of pups and dams were evaluated.

**Table 1:** Parameters of development of pups exposed to PTU or OMC in postnatal period.

**Parameters**		**Ear**		**Hair**		**Tooth**		**Eyes**	
		**Detachment**		**Growth**		**Eruption**		**Open**	
**GROUP**		**Before**	**After**	**Before**	**After**	**Before**	**After**	**Before**	**After**
		**PN4**	**PN4**	**PN5**	**PN5**	**PN10**	**PN10**	**PN14**	**PN14**
Control	Count	2	30	0	31	6	22	7	10
	% within GROUP	6.2	93.8	0	100	21.4	78.6	41.2	58.8
PTU 4 mg	Count	0	24	0	24	8	12	5	6
	% within GROUP	0	100	0	100	40	60	45.5	54.5
OMC 250 mg	Count	0	21	0	21	9	10	7	8
	% within GROUP	0	100	0	100	47.4	52.6	46.7	53.3
OMC 500 mg	Count	6	16	0	22	1	21	10	8
	% within GROUP	27.3	72.7	0	100	4.5	95.5	55.6	44.4
OMC 1.000 mg	Count	8	21	7	22	6	23	7	21^*^
	% within GROUP	27.6	72.4	24.1	75.9	20.7	79.3	25	75^*^

Postnatal day of ear detachment, hair growth, tooth eruption, and eyes open over 16 days of PTU or OMC administration for pups from both genders. Control: Corn oil (vehicle); Data are displayed as counting and percentage of counting. Animals were observed from PN1 to PN16 (N = 17). The differences were significant at p < 0.05 (^*^).

The results showed that dams from PTU (4 mg/Kg/day) and OMC (1,000 mg/Kg/day) groups as well as pups from both groups (PTU and OMC) have significantly decreased total T4 levels when measured on PN23 (Figures 1C,D). The reduction of T4 levels in the offspring was similar in the PTU and OMC group (Figure 1C).

**Figure 2:**
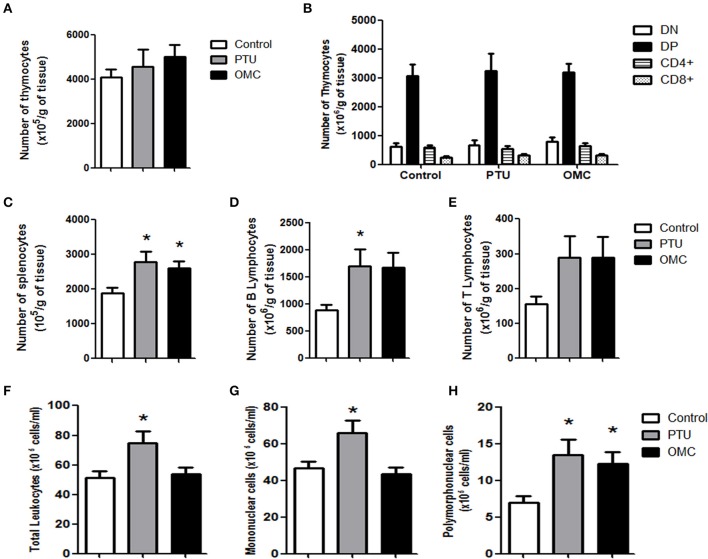
Analysis of thymus, spleen and peripheral blood of animals exposed to PTU or OMC. **(A)** The number of total thymocytes and **(B)** flow cytometry analysis of subpopulations from pups on PN23 exposed to PTU or OMC 1,000 mg/kg. **(C)** The number of total splenocytes, **(D)** B lymphocytes, and **(E)** T lymphocytes from the spleen of pups on PN23 exposed to PTU or OMC 1,000 mg/kg. *Significant difference between exposed groups and Control (*p* < 0.05). **(F)** Total leukocytes and subpopulation counts of **(G)** mononuclear cells and **(H)** polymorphonuclear cells of pups on PN23 exposed to PTU or OMC 1,000 mg/kg. (A) (*N* = 26); **(B)** (*N* = 25); **(C)** (*N* = 25); **(D)** (*N* = 7); **(E)** (*N* = 7); **(F)** (*N* = 26); **(G)** (*N* = 25); **(H)** (*N* = 25); *Significant difference between exposed groups and Control (*p* < 0.05). Error bars correspond to ± SEM.

No alteration was found in the thymus for relative weight (data not shown), the relative number of thymocytes, as well as changes in thymocyte subpopulations—double-negative (DN), double-positive (DP), CD4+ cells, and CD8+ cells (Figures 2A,B). On the other hand, the relative spleen weight (data not shown) and the relative number of splenocytes of the OMC and PTU groups significantly increased (Figure 2C). When we analyzed the numbers of B and T lymphocytes in pups' spleen, only the group exposed to PTU presented a significantly increased number of B lymphocytes (Figure 2D); however, both OMC and PTU pups presented an increased, but not statistically significant, number of T lymphocytes compared to the control group (Figure 2E). Hematological examination showed an increased level of total leukocytes counts in the PTU group, with no alteration in the OMC group compared to control (Figure 2F). The number of mononuclear cells in the PTU group increased by 1.37-fold, but no difference was observed between the OMC and control group (Figure 2G). Furthermore, a significant increase occurred in the number of polymorphonuclear (PMN) cells in the groups exposed to PTU (1.86-fold increase) and OMC (1.81-fold increase) over the control group (Figure 2H).

**Figure 3:**
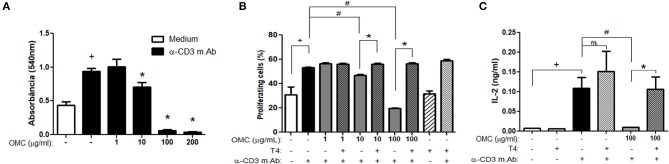
Effect of OMC on splenocyte proliferation. **(A)** Effect of different concentrations of OMC on splenocyte proliferation after anti-CD3 stimulation for 72 h. ^+^Significant difference between anti-CD3 stimulated group and non-stimulated (*p* < 0.05). *Significant difference between OMC treated group and anti-CD3 stimulated non-treated group (*p* < 0.05). **(B)** Effect of OMC treatment and T4 supplementation on splenocyte proliferation after anti-CD3 stimulation for 72 h. ^+^Significant difference between anti-CD3 stimulated group and non-stimulated (*p* < 0.05). ^#^Significant difference between OMC treated group and anti-CD3 stimulated non-treated group (*p* < 0.05). *Significant difference between OMC treated group and OMC treated and T4 supplemented group (*p* < 0.05). Error bars correspond to ±SEM. **(C)** Effect of OMC treatment and T4 supplementation on IL-2 production after anti-CD3 stimulation for 72 h. ^+^Significant difference between anti-CD3 stimulated group and non-stimulated (*p* < 0.05); ^#^Significant difference between OMC treated group and anti-CD3 stimulated non-treated group (*p* < 0.05); *Significant difference between OMC treated group and OMC treated and T4 supplemented group (*p* < 0.05); ns, not significant. Error bars correspond to ±SEM.

Since thyroid hormones may have a direct action on T lymphocytes (27) and considering the ability of the OMC to molecularly modulate the thyroid hormone receptor, we evaluated the *in vitro* effect of OMC on splenocyte activation and proliferation. As observed in Figure 3A, OMC, in concentrations ranging from 10 to 200 μg/mL, inhibited anti-CD3 induced splenocyte proliferation. To investigate whether T4 addition could block the inhibitory OMC-induced effect, splenocytes were incubated with a medium containing OMC plus T4 and then stimulated to proliferate. The incubation of splenocytes with OMC impaired cell proliferative response induced by anti-CD3 stimulation within 72 h; however, when T4 was added, the proliferative capacity of the cells increased (Figure 3B). Since interleukin (IL)-2 is a critical T-cell growth factor, we evaluated the presence of this cytokine in the supernatant from the splenocytes of the proliferation assay. The treatment of cells with OMC reduced IL-2 production and this effect was reversed when T4 was added (Figure 3C), which is in accordance with the cell proliferation data (Figure 3B). Our results indicate that the addition of T4 was able to reverse the inhibitory effect caused by the OMC.

## Discussion

Our results provide new information on the influence of the OMC on hypothyroxinemia generation and its implications on the immune system in rodents after the lactation phase.

For thyroid hormone support during lactation, the important role of breastfeeding for the passage of T4 through milk is already known (28). The levels of T4 secretion are found to be higher in lactating rats than after weaning (29). Lactating mice pups whose mothers received dietary thyroxine supplementation had higher serum T4 levels than those without this diet, and this supplementation was responsible for restoring the brain myelination process of the litter (30). Just as thyroid hormones can pass through milk, some authors have also found the presence of the UV filter OMC in breast milk (31, 32), and this has raised concern given its ability to modulate hormones. The data presented here corroborate the findings of several authors in wich OMC led to a hypothyroxinemia condition (15, 17, 32, 33). In our study, the lactating females treated with the OMC had lower T4 levels, which could transfer less T4 to their pups. However, since OMC is an inhibitor of thyroid function in the mother, it could act with the same mechanisms in the pups. The amount of T4 transferred through the milk might not be sufficient to influence T4 serum levels in the pups. It was not possible to determine whether the reduction in puppies T4 levels was due to the lower T4 intake from the mothers, or a direct effect of OMC being passed through the milk and affecting the pups' thyroid or both in a synergistic effect. In any case, the deregulation of thyroid hormones in offspring led to developmental changes similar to those observed by other authors, such as lower body weight gain in animals (9, 34). Moreover, our data indicates that OMC induced an increase in eye-opening time and was similar to results from our group using an experimental rat model (unpublished data). This developmental change in eye-opening time correlates with the formation of the central nervous system. On the other hand, even with a reduction in T4 levels, animals exposed to PTU did not have significant changes in the evaluated developmental parameters. However, even without noticeable developmental changes due to the generated hypothyroxinemia, the PTU group presented other changes that were similar to the animals exposed to the OMC, such as alterations in splenocyte counts and in subpopulations of circulating leukocytes. Another factor that may have to do with the lack of clear signs of developmental change in PTU-treated animals may be the dose used in our study. Mallela et al. (35) administrated different doses of PTU (10–100 mg/kg/day) in pregnant rats and mice. Individual fetuses did not have gross malformations from PTU treatment. Fetuses from rats presented body weights lower in the 100 mg/kg PTU treated group compared to the control group, and weights with lower PTU doses were not significantly different than the control group. In mice, PTU had no adverse effects, such as on placental weight, litter size, resorption rates, or body weights of fetuses after maternal treatment. In addition, histopathological evaluations of mice fetuses did not reveal any significant abnormalities with PTU treatment. In this sense, the lower PTU dose that we used (4 mg/kg/day) was able to induce hypothyroxinemia and correlate with the developmental findings of the study in mice by Mallela et al. (35).

Several neurodevelopmental phenomena are influenced by thyroid hormones, such as axonal and dendritic growth, migration, synaptogenesis, neural survival, oligodendrocyte proliferation and myelinization, as well as synaptic efficacy (36). Several studies have observed that hypothyroidism induced by PTU could significantly compromise rodents in memory and learning tests (37–39); moreover, changes in motor skills and learning related to the effect of the OMC have been described by Axelstad et al. (9).

Experimental and clinical evidence suggests the bidirectional interactions between the neuroendocrine system and the thymus, especially the thymus hormone activity appears to be strongly modulated by thyroid hormone signals (40, 41). Hyperthyroidism increases numbers of thymocytes, leading to thymic hyperplasia in humans, and mice exogenously injected with triiodothyronine have an increase in thymocyte proliferation and volume of the thymus (42, 43). In our study, no changes were found in total numbers of thymocytes or subpopulations of DP, DN, CD4+ and CD8+ T cells. The differences found in our results from other data in the literature could be associated with two factors, indirect exposure to endocrine disruptors through mothers and the fact that animals are in the neonatal phase. The neonatal period can generate different response pattern from those observed in adult mice. The pups from the group exposed to the OMC presented a higher number of splenocytes in contrast to unaltered number of circulating mononuclear cells in peripheral blood. The increase in splenocyte number also seems to occur with the PTU group, although this group exhibited a considerable leukocyte increase in all cell populations in the peripheral blood. Both cases of hypothyroxinemia generated by OMC or PTU did not present a reduction in leukocytes compared to the control group. Some studies found that rats and chickens with induced hypothyroidism exhibit a reduction in the number of peripheral blood lymphocytes and low responsiveness to mitogenic stimuli (44, 45). The main difference from these studies to ours is that they were performed in adult animals, and our model evaluated the neonatal period, which can lead to a difference in sensitivity and in some generated effects.

In clinical cases of hypothyroidism, PMNs have low migratory capacity when compared to healthy individuals (46); however, individuals with hyperthyroidism present PMN migratory activity similar to normal (47). A curious result was the increased number of circulating PMN cells in both PTU and OMC groups. The increased number of PMNs in the PTU group seems to be associated with a generalized increase in total leukocytes. On the other hand, the OMC group presented higher numbers of circulating PMN, with an imbalance between mononuclear and PMN cells compared to control animals. This increase in the number of circulating PMNs may be due to the accumulation of mononuclear cells in the lymphoid organs (as observed in the spleen) or a compensatory mechanism of the innate immune system to the detriment of a possible low functional activity of lymphocytes affected by low levels of T4 and/or a toxic effect of the OMC.

Some authors have already pointed to a positive correlation between hypothyroxinemia and a decrease in humoral and cell-mediated immune responses (45, 48). Our results indicate that the OMC has a direct dose-dependent blocking effect on the proliferative capacity of T lymphocytes. Curiously, T4 treatment was able to reverse the blocking effect of OMC on lymphocyte proliferation *in vitro*, suggesting that the OMC somewhat perturbs the ability of T lymphocyte activation and proliferation directly rather than by indirect mechanism via interactions between the endocrine and immune systems. Lymphocytes express thyroid receptors, produce TSH, and have enzymes for converting T4 to triiodothyronine (49–52), moreover, studies have shown that thyroid hormones modulate in an autocrine/paracrine mechanism the expression of soluble interleukin-2 receptor (sIL-2R), a marker of T lymphocyte activation (53, 54). Corroborating these data, Klecha et al. demonstrated after antigen challenge that IL-2 and interferon (IFN)-γ release increased in lymphocytes from hyperthyroid mice, while decreasing in cells from hypothyroid animals compared to control (24). We showed that diminished IL-2 production was closely related to inhibited proliferation in OMC-treated splenocytes, and the addition of T4 was able to rescue the cells from an anergic state, allowing IL-2 synthesis and proliferation. The addition of T4 alone without anti-CD3 stimulation failed to induce IL-2 synthesis and cell proliferation. Similar results were observed by Barreiro Arcos et al. (27), where thyroid hormones did not induce cell proliferation in resting T lymphocytes, but promotes cell proliferation in mitogen-stimulated T lymphocytes in a dose-dependent manner.

Surprisingly, our data show that T4 did not alter the proliferation of splenocytes stimulated with anti-CD3 antibody, unlike findings that demonstrated that T4 stimulates mitogen/antigen-induced proliferation of murine T cells (27, 55). We observed a subtle and non-significant increase in the anti-CD3 plus T4 stimulated group compared to the anti-CD3 group (58.9 + 1.8 vs. 53.1 + 1.1%), similarly, IL-2 production data correlate with these findings. The difference among our results and the findings of other groups may be linked to the proliferative stimuli used as well as the mouse strain/cell origin adopted in the studies. Both Barreiro Arcos and Varedi (27, 55) used the BALB/c inbreed strain, while we used the Swiss webster outbreed strain. This difference may imply the proliferative response pattern of the cells analyzed. Another difference was the proliferative stimulus used by us, the anti-CD3 monoclonal antibody, in contrast to the use of Concanavalin A (ConA) (27) and inactivated vírus HSV-1 (55). Anti-CD3 is specific for T lymphocyte stimulation, whereas ConA stimulates indistinctly different kinds of cells, besides, ConA and anti-CD3 have been shown to require activated differential pathways for calcium influx (56). Interestingly, Varedi et al. (55) demonstrated that the presence of T4 was ineffective to significant increase the antigen-induced proliferation of the cells from hyperthyroid animals. Also, the effect on the response to the ConA stimulus was similar to the inactivated vírus HSV-1 (55). Our results showed that the *in vitro* presence of T4 potentiated small increase in cell proliferation and IL-2 production, a similar effect was observed on splenocytes from hyperthyroid mice (55), however, in both cases, the potentiation was not significant.

Many of the immunologic effects of hypothyroidism can be reversed by administering thyroid hormones (40, 57). Data reported by other investigators demonstrated that *in vitro* T4 treatment or alterations in the normal state of the thyroid gland impacted the proliferative and cytokines production of lymphocytes (22, 25–27). Treatment of murine lymphocytes *in vitro* with T4 has been shown to increase the proliferative response to mitogens (27, 55). Klecha et al. (24) achieved similar results by reversing the inhibitory effect of PTU on lymphocyte proliferation following triiodothyronine treatment; nevertheless, the question remains whether the effects generated by the OMC *in vivo* can be reversed by treatment with thyroid hormones. At the same time, some contradictory results demonstrate that the thyroid hormones can have a negative influence on the immune response. Rats with induced hyperthyroidism presented a decrease in the peripheral blood helper/suppressor T cell ratio, while in thyroidectomized rats, this ratio increased, suggesting that thyroid hormones suppress the immune system and that thyroid hormone deficiency is associated with an increase of T lymphocyte activation (48).

In summary, this paper studied the effects of OMC using the non-autoimmune hypothyroidism model to better understand of the role of the thyroid on immune system homeostasis. Our results indicate that the OMC can act directly on lymphocyte functions; however, T4 supplementation could revive these functions. Our future research will functionally assess the *in vitro* and *in vivo* status of these different cell populations in the immune system in animals exposed to the OMC. The effects of OMC on innate and acquired immunity must be better understood.

## Data Availability Statement

All datasets generated for this study are included in the article/supplementary material.

## Ethics Statement

The experimental protocols were in accordance with the Guide for the Care and Use of Animals by the Oswaldo Cruz Foundation prepared by the Comitê de Ética no Uso de Animais (CEUA).

## Author Contributions

EG, FF, and FA conceived and designed the experiments. FF and FA wrote the paper. TP and MH performed the new experiments required by the referee. All authors performed the experiments and analyzed the data.

### Conflict of Interest

The authors declare that the research was conducted in the absence of any commercial or financial relationships that could be construed as a potential conflict of interest.
